# Castration-resistant prostate cancer: Androgen receptor inactivation induces telomere DNA damage, and damage response inhibition leads to cell death

**DOI:** 10.1371/journal.pone.0211090

**Published:** 2019-05-13

**Authors:** Vidyavathi Reddy, Asm Iskander, Clara Hwang, George Divine, Mani Menon, Evelyn R. Barrack, G. Prem-Veer Reddy, Sahn-Ho Kim

**Affiliations:** 1 Department of Urology, Vattikuti Urology Institute, Henry Ford Hospital, Detroit, MI, United States of America; 2 Department of Oncology and Hematology, Henry Ford Hospital, Detroit, MI, United States of America; 3 Department of Public Health Sciences, Henry Ford Hospital, Detroit, MI, United States of America; Thomas Jefferson University, UNITED STATES

## Abstract

Telomere stability is important for cell viability, as cells with telomere DNA damage that is not repaired do not survive. We reported previously that androgen receptor (AR) antagonist induces telomere DNA damage in androgen-sensitive LNCaP prostate cancer cells; this triggers a DNA damage response (DDR) at telomeres that includes activation of ATM, and blocking ATM activation prevents telomere DNA repair and leads to cell death. Remarkably, AR antagonist induces telomere DNA damage and triggers ATM activation at telomeres also in 22Rv1 castration-resistant prostate cancer (CRPC) cells that are not growth inhibited by AR antagonist. Treatment with AR antagonist enzalutamide (ENZ) or ATM inhibitor (ATMi) by itself had no effect on growth in vitro or in vivo, but combined treatment with ENZ plus ATMi significantly inhibited cell survival in vitro and tumor growth in vivo. By inducing telomere DNA damage and activating a telomere DDR, an opportunity to inhibit DNA repair and promote cell death was created, even in CRPC cells. 22Rv1 cells express both full-length AR and AR splice variant AR-V7, but full-length AR was found to be the predominant form of AR associated with telomeres and required for telomere stability. Although 22Rv1 growth of untreated 22Rv1 cells appears to be driven by AR-V7, it is, ironically, expression of full-length AR that makes them sensitive to growth inhibition by combined treatment with ENZ plus ATMi. Notably, this combined treatment approach to induce telomere DNA damage and inhibit the DDR was effective in inducing cell death also in other CRPC cell lines (LNCaP/AR and C4-2B). Thus, the use of ENZ in combination with a DDR inhibitor, such as ATMi, may be effective in prolonging disease-free survival of patients with AR-positive metastatic CRPC, even those that co-express AR splice variant.

## Introduction

The critical role of the androgen receptor (AR) in prostate cancer cell proliferation and survival is the enduring basis for treating advanced prostate cancer with drugs that block AR function or androgen biosynthesis [1, 2]. However, a relentless challenge is the development of resistance to these treatments, referred to as castration-resistant prostate cancer (CRPC) [3]. Remarkably, CRPC still relies on AR [4, 5], indicating a need to more fully understand the role of AR in cell survival. In this regard, we have discovered a role of AR in prostate cancer cell telomere stability [6, 7]. Notably, inactivation of this role of AR creates a DNA damage response (DDR) target, inactivation of which blocks repair and promotes cell death [8].

Telomeres are the DNA-protein structures that cap the ends of linear chromosomes, which are double-stranded DNA with a single-stranded overhang [9]. Telomeres contain many different proteins that play a role in the maintenance of telomere stability; the best characterized are the six proteins (TRF1, TRF2, Rap1, TIN2, POT1 and TPP1) that comprise a complex known as shelterin [10]. Shelterin and accessory proteins play a critical role in protecting chromosome ends from being recognized as lesions by the DNA damage machinery [11]. Inhibition or down regulation of these proteins causes telomere dysfunction, a condition in which unprotected chromosome ends resemble damaged DNA and recruit DDR factors, such as 53BP1, Mre11, and phosphorylated (activated) forms of H2AX, ATM and Rad17 [12], which in turn trigger cell cycle checkpoint activation [11, 13]. If damage can be repaired, the cell will remain viable; otherwise, cell death pathways will be activated [14]. Thus, telomere stability is important for cell viability, and telomere DNA damage creates an opportunity to inhibit telomere DNA repair and activate cell death [8].

AR antagonists induce telomere DNA damage in AR-positive LNCaP prostate cancer cells, and a DDR that has the features of a bona fide telomere DDR, namely, activation of ATM, as indicated by an increase in phosphorylated ATM (pATM) at telomeres [6–8]. Combined treatment with AR antagonist and ATM inhibitor (ATMi) increases the level of replication protein A (RPA, a marker of unrepaired single stranded DNA) at telomeres, indicating that repair of damaged telomere DNA has been blocked. This combined treatment increases the fraction of cells with sub-G_1_ DNA content (i.e., dead cells), presumably a result of cells entering mitosis with a level of telomere DNA damage that is incompatible with survival [8].

AR antagonist-induced telomere DNA damage in LNCaP prostate cancer cells appears to be mediated by telomere-associated AR, as AR-chromatin immunoprecipitate (AR-ChIP) contains telomeric DNA, isolated telomeric chromatin contains AR, and AR co-immunoprecipitates and colocalizes with shelterin proteins TIN2, TRF1 and TRF2 [6, 7]. In addition, this telomere damage is independent of AR transcriptional activity, independent of p53 status, and not due to down-regulation of telomerase [6–8]. Notably, AR antagonist does not cause genome-wide DNA damage, and agents such as etoposide that cause genome-wide DNA damage do not induce telomere DNA damage [7].

The AR antagonist bicalutamide induces telomere DNA damage in a variety of prostate cancer cells that express different forms of AR [7, 8]: LNCaP cells that express mutant AR [8], LAPC4 cells that express wild-type AR [7], and 22Rv1 cells [15] that express both full-length AR (f-AR) and a constitutively active AR splice variant, AR-V7, that lacks the ligand-binding domain [8]. The ability of AR antagonist to induce telomere DNA damage in CRPC 22Rv1 cells is intriguing because proliferation of these cells is ligand-independent and resistant to growth inhibition by AR antagonist.

Enzalutamide (ENZ) is a second-generation AR antagonist widely used to treat patients with CRPC [16], however, even tumors that initially respond eventually develop resistance [3]. The 22Rv1 human CRPC cell line is resistant to growth inhibition by ENZ; thus it is a useful model to investigate therapeutic approaches to combat ENZ resistance. The AR splice variant AR-V7 accounts for androgen-independent growth and survival of 22Rv1 cells, as knockdown of AR-V7 with siRNA inhibits survival [15]. The AR-V7 splice variant mediates ENZ resistance in 22Rv1 cells, is up-regulated during progression to CRPC in patients [15, 17], and is expressed in 19–59% of patients with AR-positive metastatic CRPC [18, 19].

Therefore, we investigated the role of full-length AR and splice variant AR-V7 in telomere stability and in the telomere DDR to AR antagonist ENZ in CRPC 22Rv1 cells. We also describe the growth inhibitory effect of combined inhibition of AR and DNA repair on CRPC 22Rv1 tumors in vivo.

## Materials and methods

### Cell culture

LNCaP (ATCC), 22Rv1 (ATCC), C4-2B (MD Anderson Cancer Center) and LNCaP/AR (a gift from Drs. Robert Reiter and Charles Sawyers) cells were grown in RPMI (Gibco BRL) containing 10% fetal bovine serum (FBS), 2.5 mM glutamine, 100 μg/ml streptomycin and 100 U/ml penicillin (complete medium) as described [7, 8]. Exponentially growing cells were treated as described in figure legends, in fetal calf serum (FCS)-containing medium. The concentration of AR antagonist that induces telomere DNA damage in prostate cancer cells is lower in charcoal-stripped fetal calf serum (CSS) than in untreated serum (FCS) (S1A Fig). However, to avoid confounding the effect of AR antagonist with the steroid hormone-depleting effect of CSS on AR activity, we use hormone-replete FCS in all experiments unless noted otherwise.

### Indirect immunofluorescence

The immunofluorescent staining of cells grown on glass slides was performed as described elsewhere [7, 8, 20]. Cells were fixed with 4% paraformaldehyde, permeabilized with 0.5% Triton X-100 and incubated at 4°C overnight with rabbit polyclonal antibodies against TIN2 [20], γ-H2AX (i.e., phosphorylated-H2AX) (Upstate), or AR (AR-N20; Santa Cruz), or mouse monoclonal antibodies against AR (AR-414; Santa Cruz), AR-V7 (AG10008; Precision) or pATM (10H11-E12, which detects phosphorylation of ATM at serine 1981; Cell Signaling). Cells were then washed and stained with FITC-labeled goat-anti-rabbit-IgG and/or Texas Red-labeled goat-anti-mouse-IgG (Molecular probes) secondary antibodies [7, 8]. Images of cells were acquired on an LSM-410 confocal microscope (Zeiss). Labeled foci were counted in enlarged photographs.

### TIF response

Telomere DNA damage (telomere dysfunction)-mediated activation of DDR signaling leads to the phosphorylation of H2AX at telomeres. Therefore, cells containing immunofluorescent foci of phosphorylated H2AX (γH2AX) that colocalizes with telomeric protein TIN2, which are referred to as *t*elomere-dysfunction *i*nduced *f*oci (TIF), are scored as a measure of DDR, as described [6, 7, 12]. Individual cells with >5 γ-H2AX foci were defined as having a TIF response, as few untreated cells have >5 TIF foci [8]. Eighty cells/treatment were counted in each of three separate experiments. For example, 85% of untreated LNCaP cells have <5 TIF foci [6]; by contrast, 85% of cells treated with AR antagonist have >5 TIF foci/cell (28% have 6–10 foci/cell, 45% have 11–20 foci/cell, and 12% have >20 foci/cell [6].

### Cell extracts and Western blotting

Cells were harvested by trypsinization, washed with PBS and suspended in Buffer A (50 mM Tris-HCl, pH 7.4, 250 mM NaCl, 0.1% Triton X-100, 5 mM EDTA, 50 mM NaF, and 0.1 mM Na_3_VO_4_) supplemented with protease inhibitor mixture (P-8340, Sigma) at a density of 2x10^7^ cells/ml as described elsewhere [7, 8, 21, 22]. Cells were then subjected twice to 30 pulses of sonication with a Branson Sonifier 250 set at output control 2 and duty cycle 20, with intermittent cooling on ice. The sonicated cell extract was cleared by centrifugation in an Eppendorf centrifuge at 12,500 rpm for 10 min [7, 8]. For Western blotting, membranes were probed with antibodies against AR (AR-N20, Santa Cruz), AR-V7 (AG10008, Precision) or GAPDH (AB2302, Millipore). Immunoreactive bands were developed using horseradish peroxidase-conjugated secondary antibodies and SuperSignal WestPico chemiluminescent substrate (Pierce), and visualized using X-ray film [7, 8].

### RT-PCR analysis

Total RNA was prepared as described [7, 8, 22]. RNA was reverse transcribed using random hexamers and oligo (dT) primer and Transcriptor Reverse Transcriptase (Roche Applied Science) according to the manufacturer's instructions [7]. PCR of cDNA was carried out using Platinum PCR SuperMix (Invitrogen) [7]. PCR primers for AR were 5’-tcagttcacttttgacctgctaa (forward) and 5’-gtggaaatagatgggcttga (reverse), PCR primers for PSA were 5’-gcacccggagagctgtgt (forward) and 5-gatcacgcttttgttcctgat (reverse) and for GAPDH were 5'-gagatccctccaaaatcaagtg (forward) and 5' ccttccacgataccaaagttgt (reverse). Cycle parameters were 94°C for 2 min, 94°C for 30 sec, 55 ^o^C for 30 sec and 68°C for 1 min. The optimal number of cycles for each gene was chosen from the linear range of amplification [23]. AR was amplified for 30 cycles, PSA for 30 cycles and GAPDH for 25 cycles [7].

### Chromatin immunoprecipitation (ChIP) analysis

ChIP analysis was performed as described elsewhere [7]. Briefly, cells harvested by scraping were washed with PBS and lysed in 1% SDS containing buffer at a density of 10^7^ cells/ml. The lysate was then sonicated using a Branson Sonifier 250 and cleared by centrifugation. The cleared lysate (0.2 ml) was diluted with 1.2 ml Buffer containing 0.01% SDS, 1.1% Triton X-100, 1.2 mM EDTA, 16.7 mM Tris-HCl, pH 8.0, and 150 mM NaCl, incubated at 4°C overnight with 5 μg antibody [IgG (Santa Cruz), AR N-20 (Santa Cruz), GR (Cell signaling), PR (Cell Signaling), RNA Pol II (Imgenex), or Rap1 (Bethyl Lab)], and the antibody-bound material was then precipitated with 30 μl protein-G Sepharose beads (Invitrogen) that had been pre-equilibrated with 30 μg bovine serum albumin (BSA) and 5 μg sheared *Escherichia coli* DNA for 30 min at 4°C. In order to isolate DNA from the ChIP pellet (ChIP DNA), cross-linking was reversed at 65°C for 4 h, treated with RNAase A and proteinase K at 37°C, and extracted with 0.5 ml phenol/chloroform/isoamylalcohol. In order to probe ChIP DNA for AR binding sites in the PSA gene, NDR G1 gene and telomeres, ChIP DNA was subjected to PCR using primers for PSA ARE III: 5’- cttctagggtgaccagagcag (forward) and 5'- gcaggcatccttgcaagatg (reverse), for NDR-G1 ARE 5’-gccacctgggtagctttgta (forward) and 5’-agaggagccgccaaattaaa (reverse) and for chromosome 17p telomeres (Chr. 17p-Tel): 5’-gaatccacggattgctttgtgtactt (sub-telomeric forward) and 5’-tgctccgtgcatctggcatc(ccctaa)_5_ (telomeric reverse). Cycle parameters for ARE III were 94°C for 2 min, 94°C for 30 sec, 55°C for 30 sec and 68°C for 1 min for 30 cycles. Cycle parameters for telomere DNA were 95°C for 3 min 94°C for 30 sec, 60°C for 30 sec, 68°C for 1 min for 35 cycles. The number of cycles for each sequence was chosen from the linear range of amplification [23].

### AR knockdown

As described elsewhere [7], exponentially growing 22Rv1 cells (1.0–2.0 x 10^5^ cells/well of a six-well plate) were transfected with 200 pmol siRNA targeting AR exon 1 (Ex1 siRNA, CAAGGAGGUUACACCAAA, to knock down both f-AR and AR-V7 [24]), AR exon 7 (Ex7 siRNA, UCAAGGAACUCGAUCGUAU, to knock down f-AR [24]), AR cryptic exon 3 (ExCE3 siRNA, GUAGUUGUGAGUAUCAUGA, to knock down AR-V7 [25]), or a control scrambled sequence (Santa Cruz), using Lipofectamine 2000 (Invitrogen) following the manufacturer’s instructions. Cryptic exon 3 [25] is also known as cryptic exon 3b [26]. Cells were processed 36 hr later for immunofluorescence staining or Western blotting. In addition, transfected cells were treated with or without ATMi KU60019 (Selleck Chemicals, TX) for an additional 24 hr prior to colony formation assay.

### Colony formation assay

This procedure is essentially as described [8, 27]. Cells (0.5–1.0 x 10^4^ cells/well of a six-well plate) were treated as described in figures for 24 hr, then washed to remove drugs and allowed to grow for 10–14 days, then fixed and stained with 0.01% crystal violet [8, 28]. The areas of stained surviving cells in each plate were photographed and measured using the ImageJ program [8, 27]. The survival fraction was plotted relative to control (vehicle) [8].

### 22Rv1 Xenografts

All experiments were performed in accordance with protocols (IACUC #1555) approved by the Institutional Animal Care and Use Committee of Henry Ford Health System. Mice were anesthetized by carbon dioxide inhalation in an approved chamber prior to cervical dislocation. In order to test the effect of AR antagonist ENZ and ATM inhibitor KU59403 (Medkoo Bioscience, NC) on 22Rv1 tumors, athymic nude mice (Charles River) were inoculated subcutaneously with 4 X 10^6^ 22Rv1 cells as described by Wu et al.[29], and when tumor size reached about 200 mm^3^, tumor-bearing mice were randomly assigned to the following 4 treatment groups: vehicle (control, 6 mice), ENZ (enzalutamide, Selleckchem, TX) alone (7 mice); ATMi KU59403 alone (7 mice); ENZ + KU59403 (6 mice). All treatments were carried out 5 days/week for 4 weeks. KU59403 (25 mg/kg) was administered twice daily by i.p. injection [30]. ENZ (50 mg/kg) was administered daily by oral gavage [31]. Tumor growth was monitored by measuring tumor volume [32] twice a week. Treatments were carried out for 4 weeks, or until tumor volume reached 2,000 mm^3^ when mice were sacrificed, and tumors were harvested.

### Immunohistochemistry

Harvested tumors were formalin-fixed, paraffin embedded, and five-micrometer sections cut to assess the activation of DDR signaling and induction of apoptosis in tumor cells. Activation of DDR signaling was assessed by immunohistochemical (IHC) staining of pATM using anti-pATM antibodies (Santa Cruz) and the IHC analysis Kit (Vector Laboratories), and apoptotic cell death was assessed by using a TUNEL assay Kit (InVitrogen), following the manufacturer’s suggested protocols.

### Statistics

Data are presented as mean +/-SD of three or more independent experiments. Statistical significance was calculated using the two-tailed Student t-test, using GraphPad Prism Software. A p-value <0.05 was considered significant.

## Results

### ENZ induces telomere damage in CRPC cells, and combining ENZ with an ATMi leads to cell death

**Fig 1:**
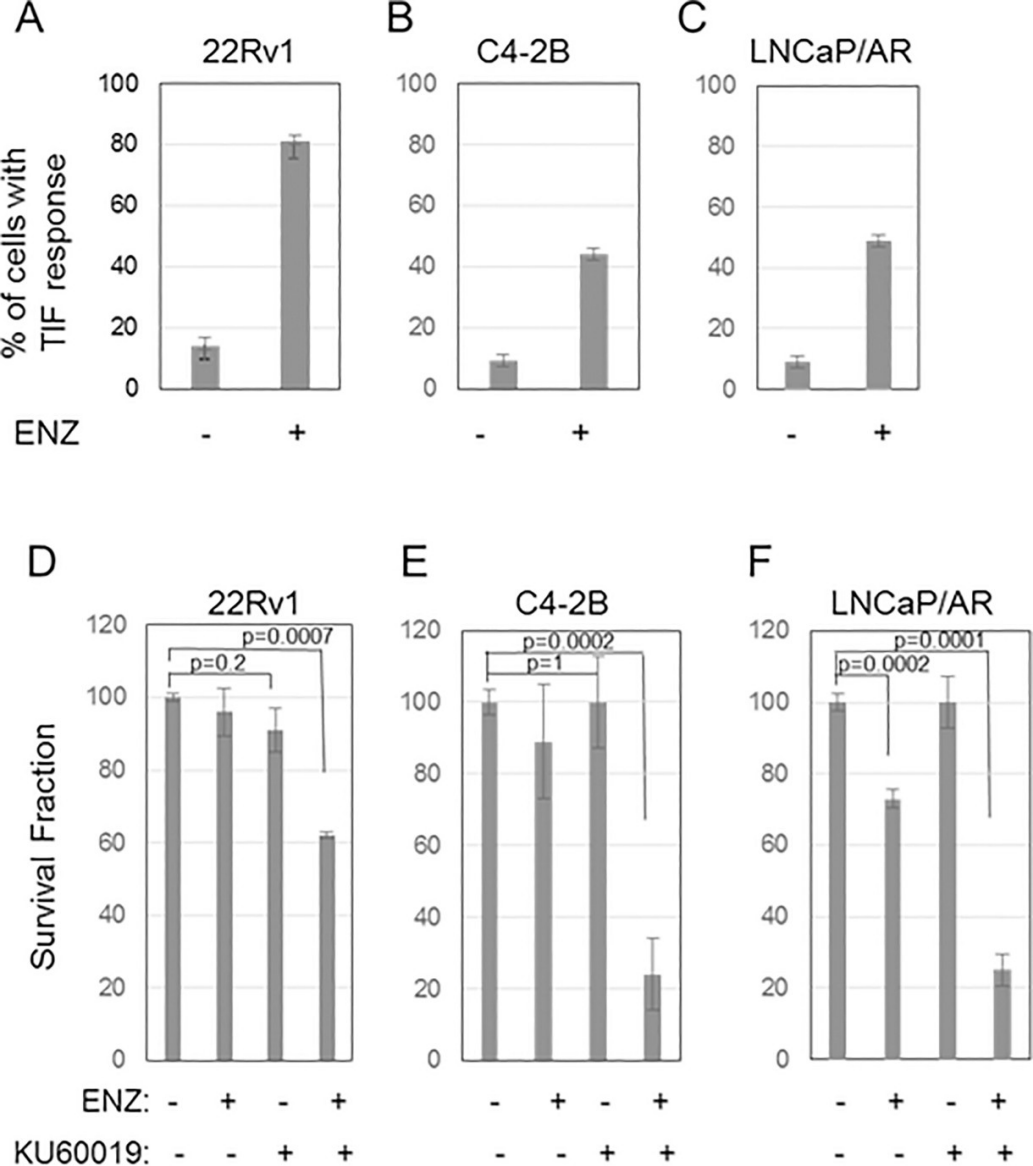
ENZ induces telomere damage, and inhibiting repair of damage with an ATMi leads to cell death, in multiple CRPC cell lines. ***(A-C)* ENZ induces telomere damage in CRPC cells.** Based on the dose-response data shown in S1A Fig, CRPC cells were treated for 24 hr with 5 μM ENZ (22Rv1) or 10 μM ENZ (C4-2B and LNCaP/AR), then labeled with antibodies to DNA damage marker γ-H2AX and the telomere marker TIN2. Colocalization of γ-H2AX and TIN2 indicate DNA damage at telomeres. Cells with a TIF response to ENZ (>5 dual-labeled foci) were counted in enlarged (1000X) photomicrographs of representative fields. Data are expressed as mean ± SD of 3 independent experiments. ***(D-E)* Combining ENZ with ATMi KU60019 leads to cell death.** 22Rv1 ***(D)***, C4-2B ***(E)***, and LNCaP/AR ***(F)*** cells were treated with 5 μM ENZ in the presence or absence of 10 μM KU60019 for 24 hr, then washed to remove drugs and allowed to grow for 14 days (colony formation assay). The survival fraction is plotted relative to vehicle-treated controls; mean ± SD of 3 independent experiments.

Despite resistance to the growth inhibitory effect of ENZ, this AR antagonist nevertheless induces telomere DNA damage in 22Rv1 CRPC cells (Fig 1A and S2A Fig), as well as in other human CRPC cell lines tested, namely, C4-2B (derived from an LNCaP xenograft propagated in castrated animals [33]) and LNCaP/AR (LNCaP cells engineered to overexpress AR [34]) (Fig 1B and 1C).

ENZ also induces activation of ATM (S2B Fig) in 22Rv1 cells, a critical mediator of the telomere DDR [13], as indicated by increased phosphorylation of ATM (pATM), the bulk of which was co-localized with TIN2 at telomeres (S2B Fig, merge image). Notably, combined treatment of CRPC cells with 1–10 μM ENZ (to induce telomere damage) plus the ATMi KU60019 (to inhibit the telomere DDR) leads to significant and substantial cell death in all three CRPC cell lines (Fig 1D–1F and S1D–S1F Fig) as it does in LNCaP cells (S1C Fig). Interestingly, ATMi by itself had no noticeable effect on AR transcriptional activity as indicated by the lack of difference in the level of expression of PSA, an AR-target gene, in 22Rv1 cells treated with ATMi vs. vector (controls) (p = 1) (S1B Fig). Thus, CRPC cells are vulnerable to treatments that target telomere stability and repair of telomere damage, though each alone has little or no effect on survival.

### Does AR splice variant AR-V7 play a role in telomere stability?

Our observation that AR antagonist induces telomere damage in AR-positive prostate cancer cells indicates a role of AR in telomere stability in these cells [6–8]. Our studies using LNCaP cells indicate that this role is mediated by a subset of AR associated with telomeres [6]. Although the AR splice variant AR-V7 cannot bind AR antagonist, AR-V7 might nonetheless play a role in telomere stability, for example, if it heterodimerized with full-length AR at telomeres. Therefore, we sought to determine whether AR-V7 is associated with telomeres in 22Rv1 cells.

**Fig 2:**
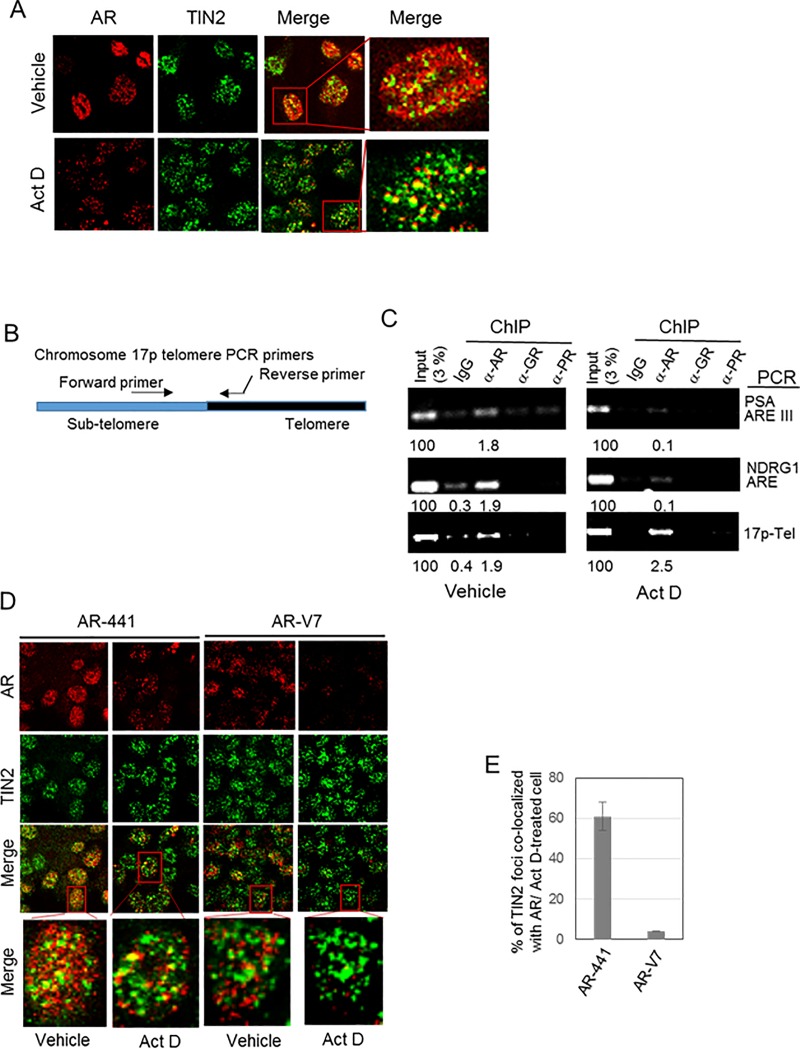
Telomere-associated AR in prostate cancer cells. ***(A-D)* Actinomycin D-resistant AR is preferentially associated with telomeres. *(A)* Actinomycin D treatment facilitates immunofluorescent identification of AR at telomeres.** LNCaP cells were treated with or without 0.5 μg/ml actinomycin D (Act D) for 4 hr, then fixed, permeabilized, and equilibrated in cytoskeleton buffer, and then subjected to dual labeling with antibodies against AR (AR-441, *red*) and TIN2 (*green*). Actinomycin D treatment greatly decreases the amount of nuclear AR without affecting TIN2. Dual-labeled foci (yellow in the *merge* panels) represent AR at telomeres, and are more evident in actinomycin D-treated cells. ***(B)*** Schematic of location of PCR primers used to amplify a region spanning the sub-telomere and telomere region of chromosome 17p. ***(C)*** A**ctinomycin D treatment decreases AR association with AREs but not with telomeres.** Chromatin was isolated from LNCaP cells that had been treated with or without actinomycin D and then fixed; chromatin immunoprecipitates (ChIPs) were prepared using antibodies against AR (N-20), GR, and PR, and analyzed by PCR for the presence of androgen response elements (AREs) of PSA and NDRG1 gene promoters, or chromosome 17p telomere DNA. The data shown is representative of 3 experiments. ***(D)* Full length AR, but not splice variant AR-V7, is associated with telomeres in 22Rv1 cells.** Immunofluorescent images of 22Rv1 cells that were treated with or without 0.5 μg/ml Act D for 4 hr, then labeled with TIN2 antibody and either antibody AR-441 (which recognizes both full-length AR and splice variant AR-V7) or an AR-V7-specific antibody are shown. The merge panels show colocalization of antibody AR441 with TIN2, but not of AR-V7 with TIN2 (both in vehicle-treated and actinomycin D-treated cells); this suggests that only full-length AR is associated with telomeres. ***(E)*** Quantitation of labeled foci in actinomycin D-treated cells in ***D***. Labeled foci were counted in twenty Act D-treated cells/group in each of three separate experiments, and expressed as the percentage of TIN2 foci colocalized with AR. Error bar represents mean ± SD, n = 3.

One approach to identifying AR association with telomeres is dual-label immunofluorescence of AR that colocalizes with TIN2 in prostate cancer cells [6]. The vast excess of AR relative to TIN2 presents a challenge. Therefore, prior to incubation with antibodies, we washed cells with cytoskeleton buffer (containing 0.1 M NaCl) plus 0.5% Triton X-100 to extract loosely bound cytoplasmic and nuclear proteins, as described by others [35, 36]. This protocol extracted a lot of loosely bound nuclear AR, as indicated by a large decrease in nuclear AR staining, and made it much easier to identify colocalization of a subset of nuclear AR with TIN2 at telomeres [6]. Nonetheless, even under these conditions, AR staining vastly exceeds TIN2 staining (Fig 2A, vehicle panels).

We previously used actinomycin D to demonstrate that the role of AR in telomere stability is independent of AR transcriptional activity, as inhibiting the expression of AR-target genes in LNCaP cells with actinomycin D does not cause telomere DNA damage [7]. This led us to hypothesize that there are two pools of nuclear AR protein in prostate cancer cells: one bound to chromatin where it functions as a transcription factor and its activity is sensitive to actinomycin D, and the other that is telomere-bound where it functions in maintaining telomere stability independent of AR transcriptional activity and is resistant to actinomycin D.

Notably, actinomycin D inhibits transcription as a result of its ability to intercalate into DNA; however, owing to differences in histone modifications and compactness of nucleosomes, actinomycin D intercalates and disrupts DNA-protein interactions at least three times more efficiently in euchromatin than in heterochromatin [37–39]. Telomeric and subtelomeric chromatin is considered heterochromatin as it is enriched in epigenetic marks that are characteristic of heterochromatin, such as H3K9me3, H4K20me3, and hypoacetylated H3 and H4 [40]. The heterochromatin state of telomeres is also evident from the presence of SIRT6, a histone deacetylase that promotes transcriptional silencing, and heterochromatin protein HP1-γ required for telomere cohesion [41, 42].

Thus, we hypothesized that actinomycin D might disrupt euchromatin-associated AR more efficiently than telomere-associated AR. We first tested this hypothesis using LNCaP cells that express only full-length AR. We treated cells with actinomycin D and then prepared them for immunolabeling of AR and TIN2. Actinomycin D treatment indeed decreased the amount of nuclear AR, but had no effect on TIN2 protein (Fig 2A). Most notably, most of the cytoskeleton buffer-resistant AR in actinomycin D-treated cells was colocalized with TIN2 (Fig 2A, Act D merge panels), providing direct evidence for the presence of a subset of AR associated with telomeres. The presence of residual, actinomycin D-resistant AR at telomeres explains why actinomycin D treatment does not cause telomere DNA damage.

We further validated the association of actinomycin D-resistant AR with telomeres by analyzing AR-chromatin immunoprecipitate (AR-ChIP) for the presence of telomere DNA, using PCR primers that amplify sequence extending from the sub-telomere region of chromosome 17p into its telomere (17p-Tel) (Fig 2B). As expected, AR-ChIP prepared from untreated LNCaP cells (Fig 2C, vehicle) contains androgen response elements (AREs) of AR-target genes PSA and NDRG1. Notably, AR-ChIP also contains telomere DNA of chromosome 17p (Fig 2C, vehicle), consistent with our previous finding of telomere repeat DNA in AR-ChIP of LNCaP cells [7].

By contrast, AR-ChIP prepared from actinomycin D-treated cells (Fig 2C, actinomycin D) had a decreased presence of AREs; this is presumably due to the ability of actinomycin D to disrupt AR binding to euchromatin and inhibit transcription. Notably, however, actinomycin D had no effect on the presence of 17p-telomere DNA in AR-ChIP (Fig 2C), affirming the association of actinomycin D-resistant AR with telomeres.

We next used actinomycin D to address the question whether the AR splice variant AR-V7 in 22Rv1 cells is associated with telomeres and whether AR-V7 plays a role in telomere stability. Dual immunofluorescence labeling of 22Rv1 cells was carried out using TIN2 antibody and either antibody AR-441 that recognizes both full-length AR and variant AR-V7, or antibody specific to AR-V7 (Fig 2D). Colocalization of the N-terminal domain of AR (using antibody AR-441) with TIN2 at telomeres was evident in vehicle-treated cells, but AR-V7 was rarely seen at telomeres (vehicle panels in Fig 2D); this suggests that it is predominantly full-length AR associated with telomeres. Treatment of 22Rv1 cells with actinomycin D and subsequent washing with cytoskeleton buffer plus 0.5% Triton X-100 allowed for a large decrease in nuclear AR staining with no change in telomere TIN2 (Fig 2D, compare vehicle vs. Act D). The predominant colocalization of AR-441 with TIN2, and relative lack of colocalization of AR-V7 with TIN2, were evident whether or not cells were pre-treated with actinomycin D (Fig 2D), but identification and quantitation of colocalized foci was facilitated by the removal of soluble and loosely bound AR (Fig 2D and 2E).

**Fig 3:**
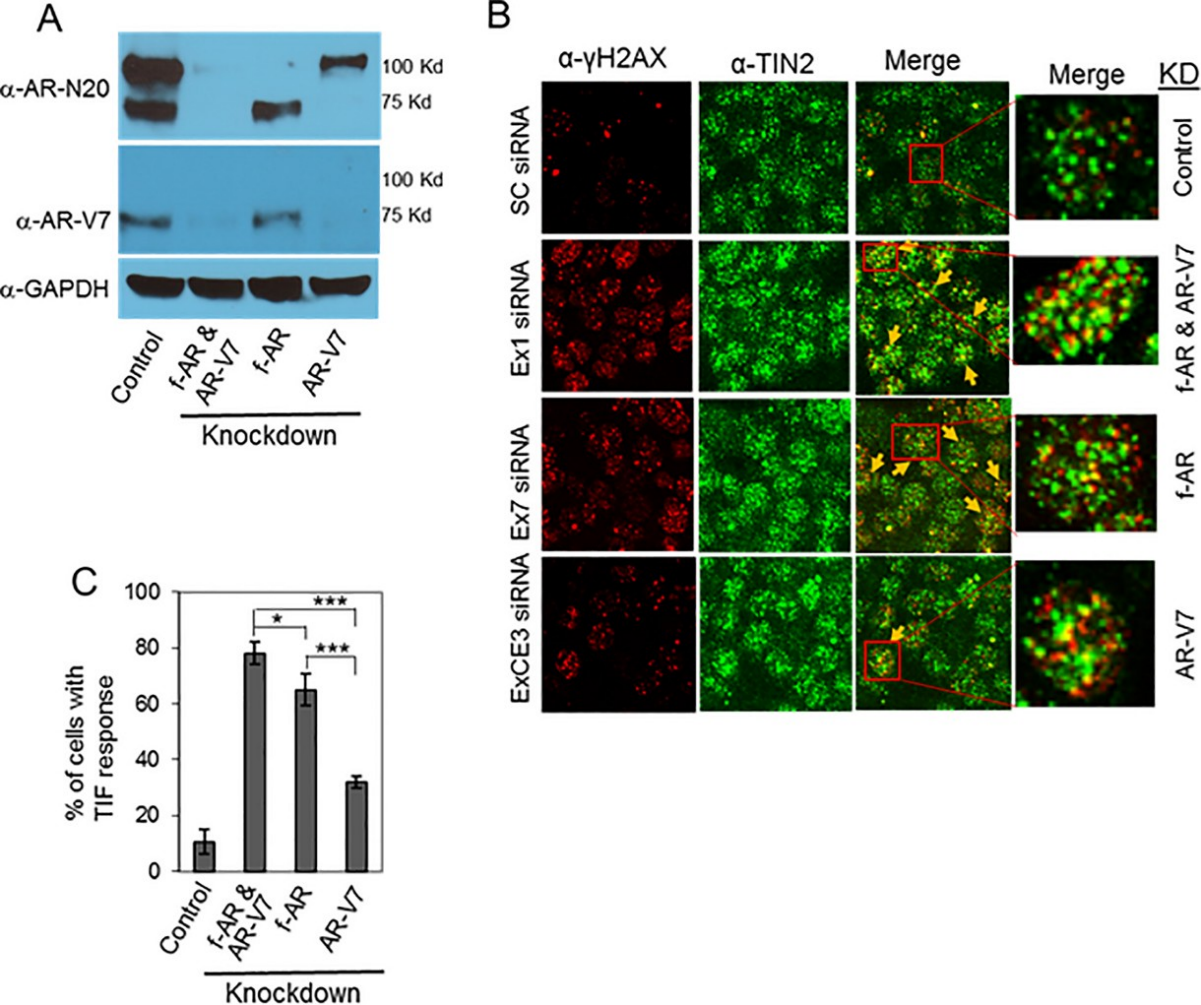
Effect of knockdown of full-length AR or AR-V7 on telomere DNA damage in 22Rv1 cells. 22Rv1 cells were transfected with siRNA targeting AR exon 1 to knockdown both full-lengh AR (f-AR) and AR-V7, AR exon 7 to knockdown only f-AR, or AR exon CE3 to knockdown only AR-V7. Scrambled siRNA was used as control. ***(A)*** Cell extracts were prepared, and western blot analysis was performed using an AR antibody (N-20) that recognizes both f-AR and AR-V7, an AR-V7-specific antibody, or a GAPDH antibody. ***(B)*** The effect of knockdown of f-AR and AR-V7, or of only AR-V7, on telomere DNA damage was assessed by dual immunofluorescent staining of the DNA damage marker γ-H2AX (red) and the telomere marker TIN2 (green). Colocalization of γ-H2AX and TIN2 (indicated by yellow arrows) is shown in the ‘merge’ panels. Higher magnification inserts of representative cells in the merge images facilitate the visualization of the presence or absence of colocalization. ***(C)*** Bar chart of the percentage of cells with a TIF response, a measure of DNA damage at telomeres. Eighty cells/treatment were counted in each of three separate experiments; mean ± SD, n = 3. *, p = 0.04; ***, p = 0.0001.

The apparent presence of a small subset of AR-V7 at telomeres in 22Rv1 cells (Fig 2F) suggests a possible role in telomere stability, perhaps via heterodimerization with full-length AR [43]. To address this question, we tested the effect of knockdown of f-AR and/or AR-V7 on telomere stability in 22Rv1 cells. We transfected cells with siRNA targeting (a) exon 1 to knockdown both f-AR and AR-V7, (b) exon 7 to knockdown only f-AR or (c) cryptic exon 3 (CE3) to knockdown only AR-V7 (Fig 3A) [15]. Knockdown of both f-AR and AR-V7 was slightly more effective than knockdown of only f-AR (p = 0.032) (Fig 3C); since cells with only f-AR knockdown still express AR-V7 (Fig 3A), a small contribution of AR-V7 to telomere stability cannot be ruled out. Knockdown of only AR-V7 caused only a low level of telomere DNA damage (Fig 3C), suggesting a small contribution of AR-V7 to telomere stability; however, we cannot rule out the possibility that this low level of telomere dysfunction was caused by the partial decrease in f-AR in these cells (Fig 3A); an effect of AR variant knockdown on f-AR has been seen also by other investigators [44]. Thus, it appears that full-length AR is the predominant form of AR at telomeres (Fig 2D and 2E), and the predominant form of AR required for telomere stability (Fig 3) in 22Rv1 cells.

**Fig 4:**
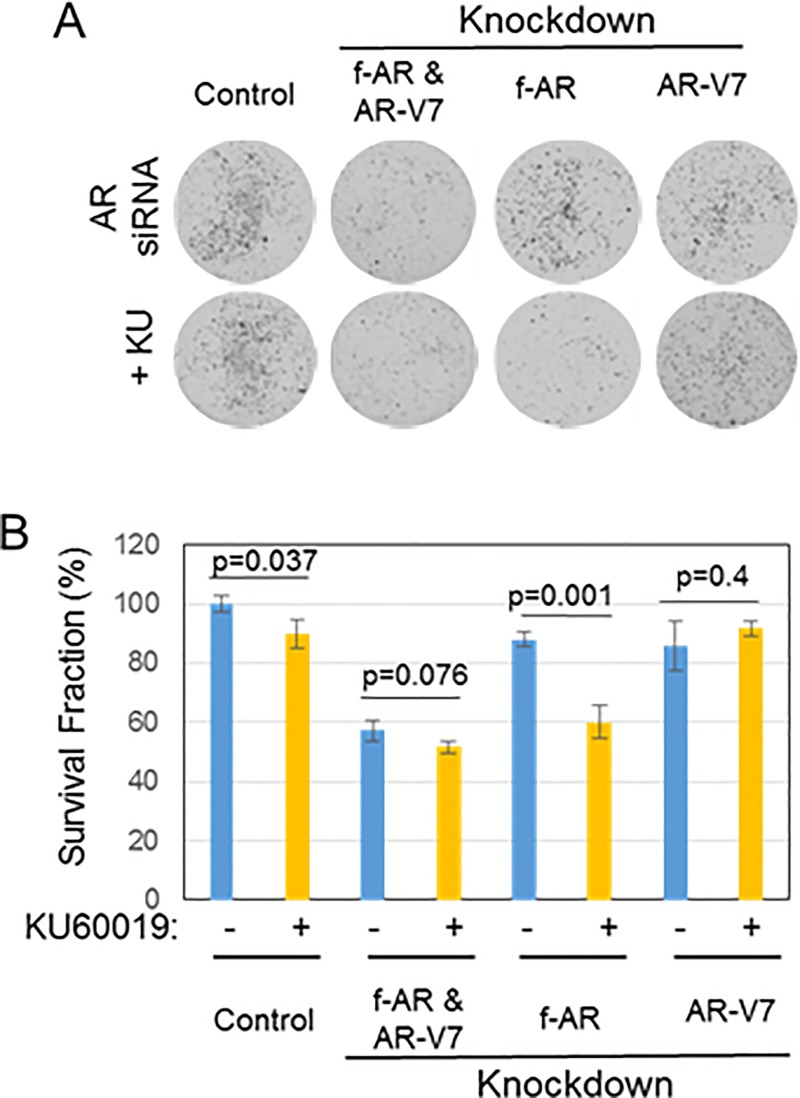
Effect of f-AR or AR-V7 knockdown in the presence of ATMi in 22Rv1 cells. **A)** 22Rv1 cells were transfected with siRNAs targeting AR exon 1 to knockdown both f-AR and AR-V7, AR exon 7 to knockdown only f-AR, or AR exon CE3 to knockdown only AR-V7. Scrambled siRNA was used as control. Transfected cells were then treated for 24 hr with or without 10 μM ATMi KU60019, then washed to remove drugs and subjected to a colony formation assay (14 day growth). **B)** Bar chart shows the survival fraction of cells from the colony formation assay, expressed relative to the survival of control cells transfected with scrambled siRNA and treated with vehicle; mean ± SD of 3 independent experiments.

We next investigated the role of full-length AR and AR splice variant AR-V7 in the cell death response to ATMi. Knockdown of full-length AR alone did not affect 22Rv1 cell survival, presumably because AR-V7 was still expressed; however, knockdown of full-length AR plus treatment with ATMi KU60019 significantly decreased cell survival (p = 0.001) (Fig 4), similar to the effect of AR antagonist ENZ plus ATMi (Fig 1D). Knockdown of AR-V7 alone did not decrease survival in hormone-replete FCS-containing medium (Fig 4) (see Materials and Methods, and S1 Fig); this is consistent with the observation that knockdown of AR-V7 decreases survival only in hormone-depleted charcoal-stripped serum (CSS)-containing medium [15]. According to Dehm and colleagues [15], knockdown of AR-V7 in the presence of androgen restores androgen responsiveness; with AR-V7 knocked down, 22Rv1 cells grow in response to androgen and this effect is blocked by AR antagonist, presumably via full-length AR. Thus, inactivation of full-length AR, when AR-V7 is knocked down, decreases survival of 22Rv1 cells [15]; this is similar to the effect of knockdown of full-length AR in other CRPC cells that express only full-length AR and rely on AR for survival [4, 5]. This likely explains why knockdown of both full-length AR and variant AR-V7 decreased survival and why survival was not further reduced by additional treatment with ATMi (Fig 4). As siRNA is not yet a reliable method for treatment in vivo, therefore we next tested the effect of combined antagonism of full-length AR plus inhibition of ATM on CRPC tumor growth in vivo.

### Treatment with ENZ plus ATMi suppresses CRPC 22Rv1 tumor growth in vivo

**Fig 5:**
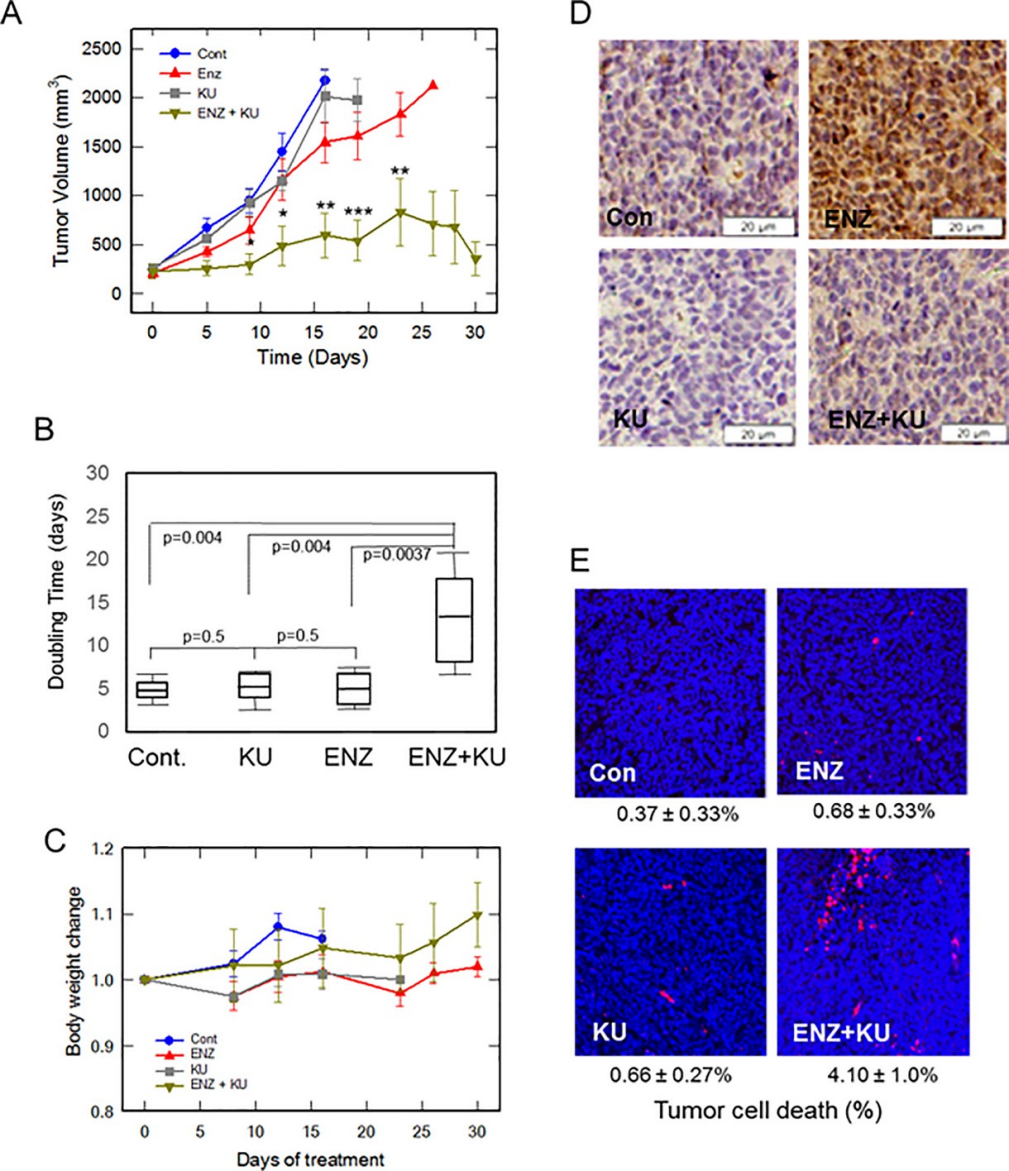
Combined treatment with AR antagonist ENZ plus ATMi inhibits growth of CRPC 22Rv1 xenograft tumors in mice that are resistant to each drug alone. 22Rv1 tumor-bearing athymic nude mice were randomly assigned to vehicle control (Cont), ENZ, ATMi KU59403 (KU), or combined ENZ+KU treatment for 4 weeks. **A)** Tumor size over time is presented as mean tumor volume (mm^3^) of each treatment group (n = 6 or 7 mice/group). Error bars represent standard deviation. Statistical analysis was performed for comparison between ENZ alone or KU alone and KU+ENZ treatments: *, p<0.05; **, p<0.001; ***, p<0.0001. **B)** The doubling time of each tumor was calculated from a plot of log tumor volume vs. time. Doubling times of each treatment group are presented as Box-Whisker plots; horizontal lines represent mean, first and third quartiles, and whiskers represent the minimum and maximum doubling time of each group. The doubling time (days, mean ± SEM) of each group was: Control, 4.94 ± 1.0; KU, 5.50 ± 1.36; ENZ, 5.46 ± 1.36; ENZ+KU, 12.71 ± 4.38. P values are shown in the chart. **C)** Body weight of mice during the treatment period, relative to day 0 of treatment of each group. **D)** ENZ induces ATM activation in 22Rv1 xenograft tumors. Immunostaining of pATM is shown in a representative 22Rv1 xenograft tumor section from each treatment group. **E)** Evaluation of cell death in serial sections of 22Rv1 tumors. TUNEL assay to detect cell death was performed as described in the manufacturer’s protocol (InVitrogen). Images show ~1, 000 cells (blue) in a representative tumor tissue section from each treatment group. Cell death was analyzed by counting the percentage of cells that were dead (red) in each image.

We treated established CRPC 22Rv1 tumors with vehicle, ENZ alone, ATMi alone, or ENZ in combination with ATMi (Fig 5). We used the ATMi KU59403, which has been shown previously to have favorable pharmacokinetic properties and bioavailability in nude mice [30]. Importantly, when administered by itself, KU59403 has no cytotoxic effects on vital organs or on body weight of mice [30].

Combined treatment of CRPC 22Rv1 tumor-bearing mice with ENZ plus ATMi dramatically inhibited tumor growth compared to the other treatments (Fig 5A; individual tumor growth curves are shown in S3B Fig). Not surprisingly, ENZ alone or ATMi alone had no significant effect on tumor growth (Fig 5A and S3 Fig). Plotting the log of tumor volume vs. time allowed us to calculate tumor doubling times, which showed that only combined treatment with ENZ plus ATMi KU59403 slowed tumor growth (Fig 5B). Notably, of 6 mice in the combined treatment group, tumor in 1 mouse became undetectable by day 9, and tumors in 2 other mice started to decrease in size beginning at day 20 (S3B Fig). Kaplan-Meier analysis revealed a significant survival benefit in the combined treatment group (S4 Fig and S1 Table). Also, consistent with earlier reports [30], ATMi KU59403 was safe *in vivo* as it had no detrimental effect on body weight when administered alone or in combination with ENZ, compared to controls (Fig 5C).

Notably, treatment of 22Rv1 tumor-bearing mice with ENZ alone activated ATM (pATM) in vivo (Fig 5D), as it did in vitro (S2B Fig), but as expected did not cause cell death (Fig 5E, TUNEL assay). Combined treatment with ENZ plus ATMi suppressed tumor growth (Fig 5A and S3 Fig) and increased cell death (Fig 5E).

## Discussion

The expression of constitutively active AR splice variants such as AR-V7, which lacks the ligand binding domain, is considered an important mechanism of AR antagonist-resistant growth of metastatic CRPC [15, 17]. However, because full-length AR is expressed in 80–100% of AR-positive CRPC metastases [18, 45], and because full-length AR, not AR-V7, is associated with telomeres and is critical for telomere stability, CRPC metastases are expected to be vulnerable to telomere DNA damage by AR antagonist, and activation of a telomere DDR that can be inhibited by an ATMi to promote cell death, as demonstrated in CRPC 22Rv1 cells and tumors, and in CRPC cell lines C4-2B and LNCaP/AR (this study). Combined treatment with ENZ plus ATMi suppressed tumor growth (Fig 5A and S3 Fig) and increased cell death (Fig 5E), presumably a result of ATMi blocking DNA repair in cells with a DDR and activated pATM, so that cells with too much damage undergo cell death [8, 14, 46].

Thus, our data suggest that the use of ENZ in combination with a DDR inhibitor, such as an ATMi, may be effective in prolonging disease-free survival of patients with AR-positive mCRPC, even the 19–59% that co-express an AR splice variant [18, 45].

The AR is a well-characterized transcription factor that regulates the expression of many genes [19], including many that play a role in DNA repair [47, 48]. In those studies, the growth inhibitory effect of ionizing radiation-induced genome-wide DNA damage was enhanced by AR inactivation, and this enhancement was inferred to be the result of decreased expression of DNA repair genes [47, 48]. By contrast, our data indicate that AR antagonist induces telomere DNA damage independent of an effect on AR transcriptional activity [7]. In addition, AR inactivation does not cause genome-wide DNA damage [6, 47], but by inducing telomere DNA damage and activating a telomere DDR, an opportunity to inhibit DNA repair is created, the consequence of which is to promote cell death, as genome integrity is required for survival. Notably, f-AR, but not AR-splice variant AR-V7, seems to be responsible for causing telomere DNA damage. As shown in Fig 3C, KD of both f-AR and AR-V7 (by targeting exon 1 with siRNA) or only f-AR (by targeting exon 7) caused telomere dysfunction, as measured by TIF response, in 70–80% of cells. By contrast, about 30% of cells exhibited telomere dysfunction when only AR-V7 was down-regulated (by targeting exon 3b); some of this effect may be due to a partial decrease in f-AR in these cells (see Fig 3A, compare lanes AR-V7 vs. control). However, whether knockdown of AR decrease f-AR due to blocking the f-AR/AR-V7 heterodimer [43] or due to the non-specific effect of KD of f-AR by exon 3b siRNA remains to be determined.

When applied to the treatment of CRPC 22Rv1 cells and tumors, combined treatment with AR antagonist plus ATMi significantly inhibited growth, whereas each drug alone was ineffective. This feature fits the criterion for synthetic lethality [49], although incomplete tumor elimination in our study suggests the need to target additional pathways or other components of the telomere DDR and repair pathway.

There is growing interest in combined targeting of AR and DNA repair in CRPC, using ENZ or androgen ablation plus an inhibitor of poly (ADP-ribose) polymerase (PARP) [50, 51] or an inhibitor of both Chk1 and Chk2 [52]. Notably, these other studies focus on inhibiting genome-wide DNA repair, whereas our own studies focus on inhibiting the repair of telomere DNA damage. Cesare et al [53] have reported that the telomere DDR is functionally distinct from the genomic DDR.

Many cell types, including prostate cancer, use AR to modulate specific gene expression, but prostate cancer cells uniquely require AR to regulate cell proliferation and cell survival. Similarly, most cells do not need AR for telomere stability, but our studies have shown a critical role of AR in telomere stability in prostate cancer cells [6]. Our data indicate that this role is mediated by a subset of AR associated with telomeres ([6] and this study), although it is not yet clear how AR is tethered to telomeres. Immunofluorescent colocalization of AR with telomere protein TIN2 was seen clearly after pretreating cells with actinomycin D and washing with cytoskeleton buffer to extract soluble nuclear AR (Fig 2). Thus, actinomycin D treatment may be a useful tool for studying telomere-associated AR.

In summary, we have demonstrated that CRPC 22Rv1 tumor growth is resistant to AR antagonist or ATMi as monotherapies, but is significantly inhibited by combined treatment. CRPC cells that express both full-length AR and splice variant AR-V7 and are not growth inhibited by AR antagonist are inferred to depend on, and are said to be driven by AR-V7; but, ironically, it is their expression of full-length AR that makes them sensitive to growth inhibition by combined treatment with ENZ plus ATMi.

## Supporting information

S1 Fig **Dose-response effect of enzalutamide (ENZ) on telomere DNA damage (A), lack of effect of ATM inhibitor on AR-target gene expression (B), and effect of ENZ + ATM inhibitor KU60019 on cell survival (C-F) in prostate cancer cells. *(A)* The concentration of AR antagonist enzalutamide (ENZ) that induces telomere DNA damage in prostate cancer cells is lower in charcoal-stripped serum (CSS) than in untreated serum (FCS).** Exponentially growing LNCaP cells were treated as indicated for 24 hr, in either FCS-containing medium (hormone-replete) or CSS-containing medium (hormone-depleted). To prepare cells for treatment with AR antagonist ENZ in CSS medium, exponentially growing LNCaP cells in FCS medium were washed twice with phenol red-free RPMI medium (Thermo Fisher Scientific) for 1 hr, and incubated in phenol red-free RPMI medium supplemented with 10% CSS (InVitrogen) for 26 hr prior to treatment with AR antagonists. After 24-hr treatment with 1–10 μM AR antagonist, cells were labeled with antibodies to γ-H2AX (marker of DNA damage) and TIN2 (telomere specific protein), and cells with a TIF response (>5 dual-labeled foci/cell) were counted. Data are expressed as mean ± SD of 3 independent experiments. The concentration of ENZ that induces telomere DNA damage in LNCaP cells was lower in hormone-depleted CSS medium (1 μM) than in hormone-replete FCS medium (10 μM). ***(B)* ATMi (KU60019) has no effect on expression of the AR target gene PSA.** 22Rv1 cells were treated without or with 10 μM KU60019 for 24 hr. PSA and GAPDH mRNA levels were assayed by RT‐PCR. ***(C-F)* Dose-response effect of ENZ in the absence vs. presence of 10 μM ATMi on survival of androgen-sensitive *LNCaP* and CRPC 22Rv1, C4-2B, and LNCaP/AR cells.** Cells were treated for 24 hr as indicated, then washed to remove drugs and allowed to grow for 14 days (colony formation assay). The survival fraction is plotted relative to vehicle-treated controls; mean ± SD of 3 independent experiments.
(TIF)

S2 Fig **ENZ induces telomere DNA damage (A) and activates ATM at telomeres (B) in CRPC cells. *(A)*** 22Rv1 cells were treated without (control, Con) or with 5 μM ENZ in FCS-containing medium for 6 hr, then labeled with antibodies to DNA damage marker γ-H2AX (red) and the telomere marker TIN2 (green). Dual-labeled foci (indicated by yellow) are shown in the ‘merge’ panel, indicating DNA damage at telomeres of ENZ-treated 22Rv1 cells. ***(B)*** 22Rv1 cells were treated with or without 5 μM ENZ for 6 hr, then labeled with antibodies to phosphorylated ATM (pATM, red) and TIN2 (green). Colocalization of pATM (activated ATM) and TIN2 is shown in the ‘merge’ panels, indicating the presence of activated ATM at telomeres of ENZ-treated 22Rv1 cells. Higher magnification inserts of representative cells in the merge images in ***A*** and ***B*** facilitate the visualization of the presence or absence of colocalization.
(TIF)

S3 Fig Combined treatment with AR antagonist plus ATMi inhibits growth of CRPC 22Rv1 xenograft tumors in mice that are resistant to each drug alone. These data supplement the data shown in Fig 5. In this Figure, tumor volumes were normalized to the start of treatment on day 0, and are shown as fold change. **A)** Data for each group are shown as mean ± SEM. *, p<0.05; **, p<0.001; ***, p<0.0001. **B)** Growth curves are shown for each tumor.
(TIF)

S4 Fig Kaplan-Meier survival analysis of 22Rv1 xenograft mice treated with AR antagonist plus ATMi. Survival was defined as the number of days until sacrifice, when tumor size was ~2,000 mm^3^. Time to sacrifice was not adjusted for differences in tumor size at the start of treatment.
(TIF)

S1 Table Median days to sacrifice (tumor volume ~2000 mm^3^). (DOCX)
