# Supplementary figures and images for: Castration-resistant prostate cancer: Androgen receptor inactivation induces telomere DNA damage, and damage response inhibition leads to cell death
Source: PLoS One. 2019 May 13;14(5):e0211090. doi: 10.1371/journal.pone.0211090 (PMC6513077; doi:10.1371/journal.pone.0211090)

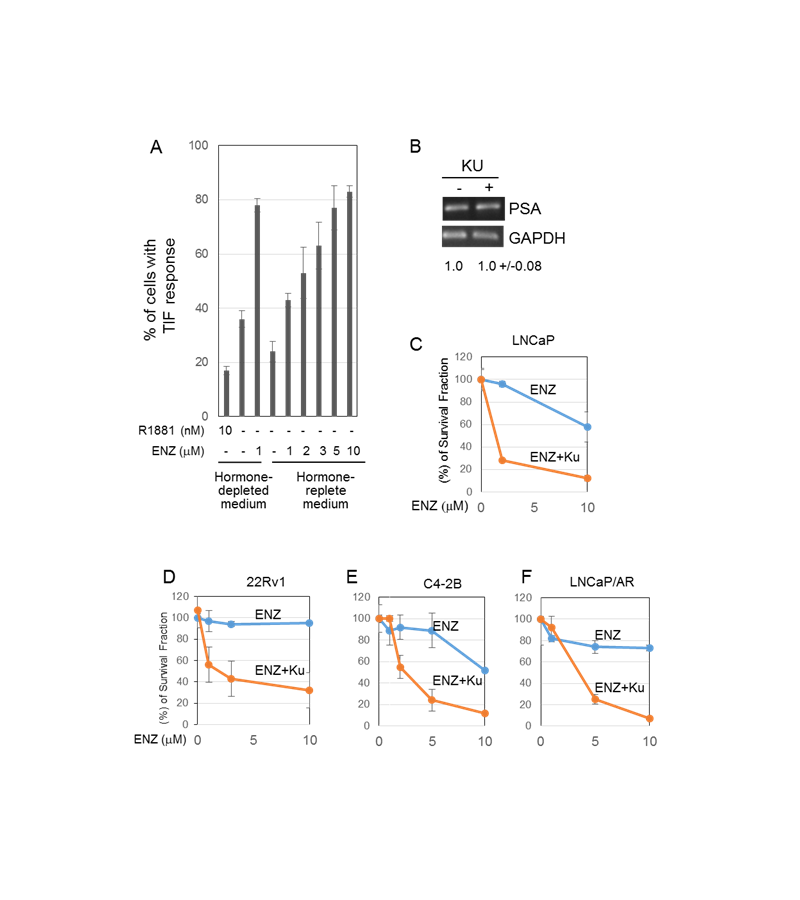

Supplement: S1 Fig — Dose-response effect of enzalutamide (ENZ) on telomere DNA damage (A), lack of effect of ATM inhibitor on AR-target gene expression (B), and effect of ENZ + ATM inhibitor KU60019 on cell survival (C-F) in prostate cancer cells. (A) The concentration of AR antagonist enzalutamide (ENZ) that induces telomere DNA damage in prostate cancer cells is lower in charcoal-stripped serum (CSS) than in untreated serum (FCS). Exponentially growing LNCaP cells were treated as indicated for 24 hr, in either FCS-containing medium (hormone-replete) or CSS-containing medium (hormone-depleted). To prepare cells for treatment with AR antagonist ENZ in CSS medium, exponentially growing LNCaP cells in FCS medium were washed twice with phenol red-free RPMI medium (Thermo Fisher Scientific) for 1 hr, and incubated in phenol red-free RPMI medium supplemented with 10% CSS (InVitrogen) for 26 hr prior to treatment with AR antagonists. After 24-hr treatment with 1–10 μM AR antagonist, cells were labeled with antibodies to γ-H2AX (marker of DNA damage) and TIN2 (telomere specific protein), and cells with a TIF response (>5 dual-labeled foci/cell) were counted. Data are expressed as mean ± SD of 3 independent experiments. The concentration of ENZ that induces telomere DNA damage in LNCaP cells was lower in hormone-depleted CSS medium (1 μM) than in hormone-replete FCS medium (10 μM). (B) ATMi (KU60019) has no effect on expression of the AR target gene PSA. 22Rv1 cells were treated without or with 10 μM KU60019 for 24 hr. PSA and GAPDH mRNA levels were assayed by RT‐PCR. (C-F) Dose-response effect of ENZ in the absence vs. presence of 10 μM ATMi on survival of androgen-sensitive LNCaP and CRPC 22Rv1, C4-2B, and LNCaP/AR cells. Cells were treated for 24 hr as indicated, then washed to remove drugs and allowed to grow for 14 days (colony formation assay). The survival fraction is plotted relative to vehicle-treated controls; mean ± SD of 3 independent experiments. (TIF) [file pone.0211090.s001.tif]

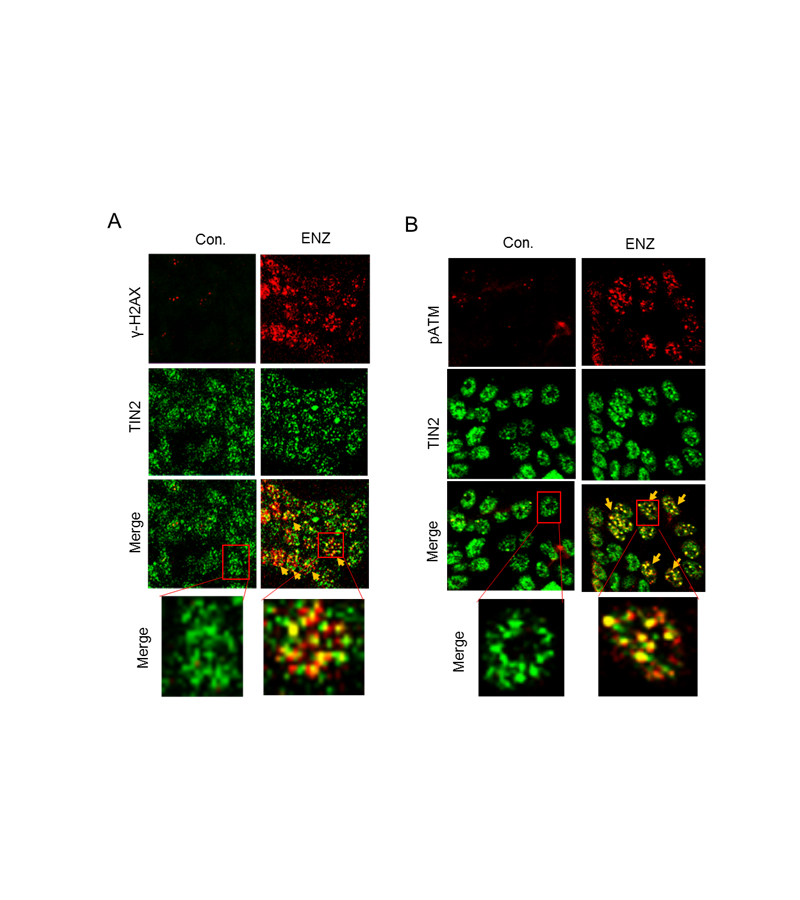

Supplement: S2 Fig — ENZ induces telomere DNA damage (A) and activates ATM at telomeres (B) in CRPC cells. (A) 22Rv1 cells were treated without (control, Con) or with 5 μM ENZ in FCS-containing medium for 6 hr, then labeled with antibodies to DNA damage marker γ-H2AX (red) and the telomere marker TIN2 (green). Dual-labeled foci (indicated by yellow) are shown in the ‘merge’ panel, indicating DNA damage at telomeres of ENZ-treated 22Rv1 cells. (B) 22Rv1 cells were treated with or without 5 μM ENZ for 6 hr, then labeled with antibodies to phosphorylated ATM (pATM, red) and TIN2 (green). Colocalization of pATM (activated ATM) and TIN2 is shown in the ‘merge’ panels, indicating the presence of activated ATM at telomeres of ENZ-treated 22Rv1 cells. Higher magnification inserts of representative cells in the merge images in A and B facilitate the visualization of the presence or absence of colocalization. (TIF) [file pone.0211090.s002.tif]

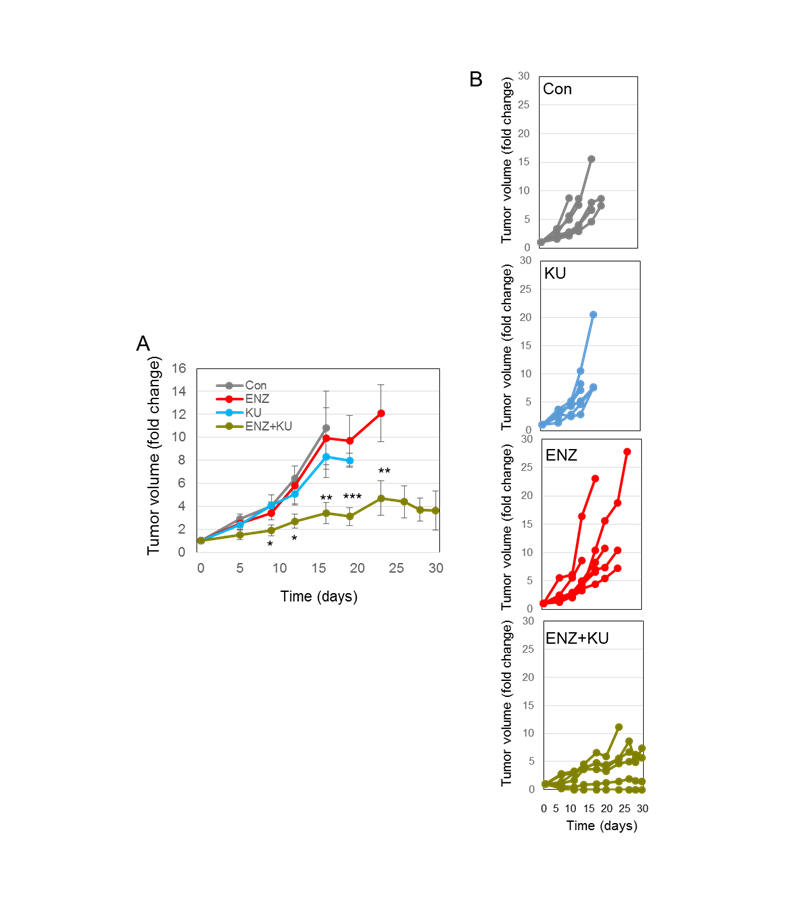

Supplement: S3 Fig — These data supplement the data shown in Fig 5. In this Figure, tumor volumes were normalized to the start of treatment on day 0, and are shown as fold change. A) Data for each group are shown as mean ± SEM. *, p<0.05; **, p<0.001; ***, p<0.0001. B) Growth curves are shown for each tumor. (TIF) [file pone.0211090.s003.tif]

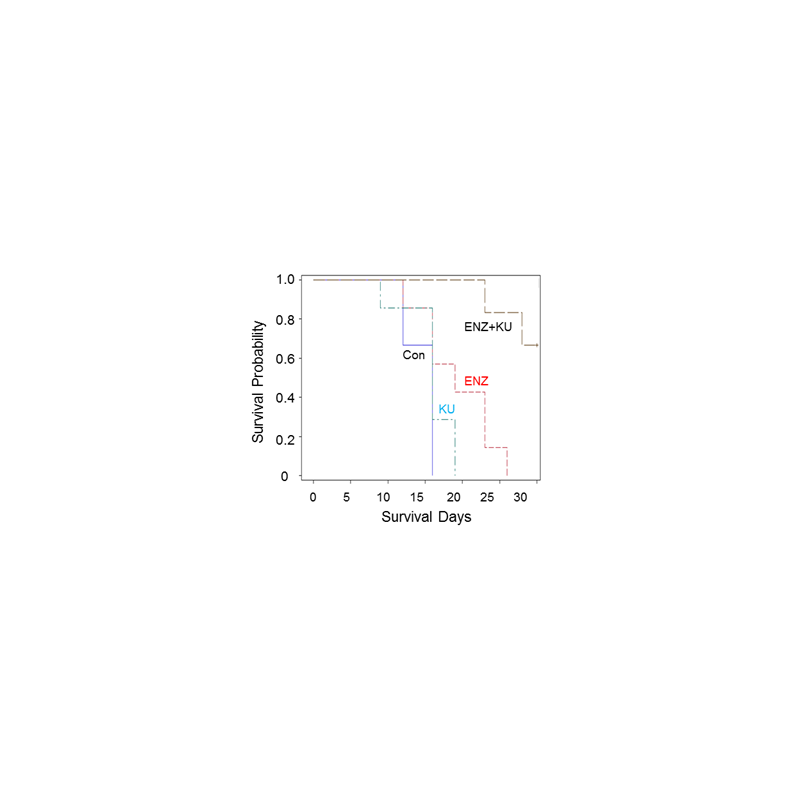

Supplement: S4 Fig — Survival was defined as the number of days until sacrifice, when tumor size was ~2,000 mm3. Time to sacrifice was not adjusted for differences in tumor size at the start of treatment. (TIF) [file pone.0211090.s004.tif]
