# Supplementary material for: Castration-resistant prostate cancer: Androgen receptor inactivation induces telomere DNA damage, and damage response inhibition leads to cell death
Source: PLoS One. 2019 May 13;14(5):e0211090. doi: 10.1371/journal.pone.0211090 (PMC6513077; doi:10.1371/journal.pone.0211090)
Supplement: S1 Table — (DOCX) [file pone.0211090.s005.docx]

**S1 Table. Median Days to Sacrifice (tumor volume ~2000 mm^3^)**

| Treatment |  | Log rank p-value | | |
| --- | --- | --- | --- | --- |
|  | Median (95% CI) | vs Control | vs ENZ | vs KU |
| Control | 16 (12, 16) |  |  |  |
| ENZ | 19 (12, 23) | 0.882 |  |  |
| KU59403 (KU) | 16 (9, 19) | 0.043 | 0.496 |  |
| ENZ+KU | >30 (23, >30) | <0.001 | 0.001 | <0.001 |

For each treatment group, the median and 95% confidence interval for time to tumor volume ~2000 mm^3^ and log rank p-values were computed using the SAS procedure PROC LOGISTIC. Time to sacrifice was not adjusted for differences in tumor size at the start of treatment.
